# An Assessment of Different Decision Support Software from the Perspective of Potential Drug–Drug Interactions in Patients with Chronic Kidney Diseases

**DOI:** 10.3390/ph17050562

**Published:** 2024-04-28

**Authors:** Muhammed Yunus Bektay, Aysun Buker Cakir, Meltem Gursu, Rumeyza Kazancioglu, Fikret Vehbi Izzettin

**Affiliations:** 1 Department of Clinical Pharmacy, Istanbul University-Cerrahpasa, Istanbul 34500, Turkey; 2 Department of Clinical Pharmacy, Bezmialem Vakif University, Istanbul 34093, Turkey; 3 Department Nephrology, Bezmialem Vakif University, Istanbul 34093, Turkey

**Keywords:** drug–drug interaction, clinical decision support software, chronic kidney disease, clinical pharmacist, pharmaceutical care

## Abstract

Chronic kidney disease (CKD) is a multifaceted disorder influenced by various factors. Drug–drug interactions (DDIs) present a notable risk factor for hospitalization among patients with CKD. This study aimed to assess the frequency and attributes of potential DDIs (pDDIs) in patients with CKD and to ascertain the concordance among different Clinical Decision Support Software (CDSS). A cross-sectional study was conducted in a nephrology outpatient clinic at a university hospital. The pDDIs were identified and evaluated using Lexicomp^®^ and Medscape^®^. The patients’ characteristics, comorbidities, and medicines used were recorded. The concordance of different CDSS were evaluated using the Kendall W coefficient. An evaluation of 1121 prescribed medications for 137 patients was carried out. The mean age of the patients was 64.80 ± 14.59 years, and 41.60% of them were male. The average year with CKD was 6.48 ± 5.66. The mean number of comorbidities was 2.28 ± 1.14. The most common comorbidities were hypertension, diabetes, and coronary artery disease. According to Medscape, 679 pDDIs were identified; 1 of them was contraindicated (0.14%), 28 (4.12%) were serious-use alternative, and 650 (9.72%) were interventions that required closely monitoring. According to Lexicomp, there were 604 drug–drug interactions. Of these interactions, 9 (1.49%) were in the X category, 60 (9.93%) were in the D category, and 535 (88.57%) were in the C category. Two different CDSS systems exhibited statistically significant concordance with poor agreement (W = 0.073, *p* < 0.001). Different CDSS systems are commonly used in clinical practice to detect pDDIs. However, various factors such as the operating principles of these programs and patient characteristics can lead to incorrect guidance in clinical decision making. Therefore, instead of solely relying on programs with lower reliability and consistency scores, multidisciplinary healthcare teams, including clinical pharmacists, should take an active role in identifying and preventing pDDIs.

## 1. Introduction

Drug–drug interactions (DDIs) contribute to 5–14% of adverse drug reactions in hospitalized patients [1]. They pose a significant risk for hospitalization, especially among elderly ambulatory patients [2]. Clinical decision support software (CDSS) systems have shown to enhance patient safety, care quality, and efficiency [3]. A CDSS assists prescribers by providing dosing guidance and alerts for duplicate therapies, drug allergies, and potential drug–drug interactions (pDDIs) [4]. The timely identification of DDIs is thus crucial for ensuring safe pharmacotherapy. CDSS systems have demonstrated efficacy in promptly detecting pDDIs [5].

Identifying DDIs involves considering various factors, including healthcare professionals’ expertise and the clinical importance of DDIs. Typically, physicians receive limited pharmacology training in medical school, relying on experiential learning thereafter. The introduction of handheld technology has aided physicians, medical residents, pharmacists, and students in enhancing DDI assessments, augmenting drug knowledge, and minimizing reliance on other drug references [6]. Depending solely on a single proprietary source for determining clinical significance may be misleading. Variations in knowledge-based composition and severity rankings across CDSS lead to a lack of standardization in DDI ratings and classifications [7]. Different drug interaction softwares reveal discrepancies in rating systems that used to describe drug interactions, further underscoring the need for standardization [8]. A comparative analysis of these systems demonstrated that 2.2% of the most severe interactions were identified in different compendia, while only 10% of the listed drug interactions were found across different software platforms [7,8].

Chronic kidney disease (CKD) is a complex disorder influenced by various factors, and comorbidities often manifest early in the course of the disease. Non-modifiable risk factors, such as age, gender, race, diabetes mellitus, and genetic predisposition, contribute to CKD, while modifiable risk factors like hypertension, proteinuria, and metabolic factors also play a significant role [9]. Given the high prevalence of comorbidities among patients with CKD, which include both underlying diseases and adverse effects stemming from impaired kidney function, management typically necessitates multiple medications. Polypharmacy, which is frequently encountered in patients with CKD, is an important risk factor for DDIs.

Individuals diagnosed with CKD often contend with a multitude of comorbidities, encompassing both primary conditions and complications arising from impaired renal function. These comorbidities commonly include hypertension, diabetes, cardiovascular disease (CVD), anemia, and bone and mineral disorders [10,11,12]. Managing these conditions necessitates the administration of multiple medications to alleviate symptoms and slow disease progression. However, this polypharmacy regimen concurrently increases the risk of encountering drug interactions.

The primary objective of this study was to assess and compare the clinical decision support systems and interaction checker applications provided by Lexicomp and Medscape. Specifically, this study aimed to evaluate the frequency and characteristics of pDDIs in patients with CKD and determine the concordance between different clinical decision support systems and interaction checker applications.

## 2. Results

**Figure 1:**
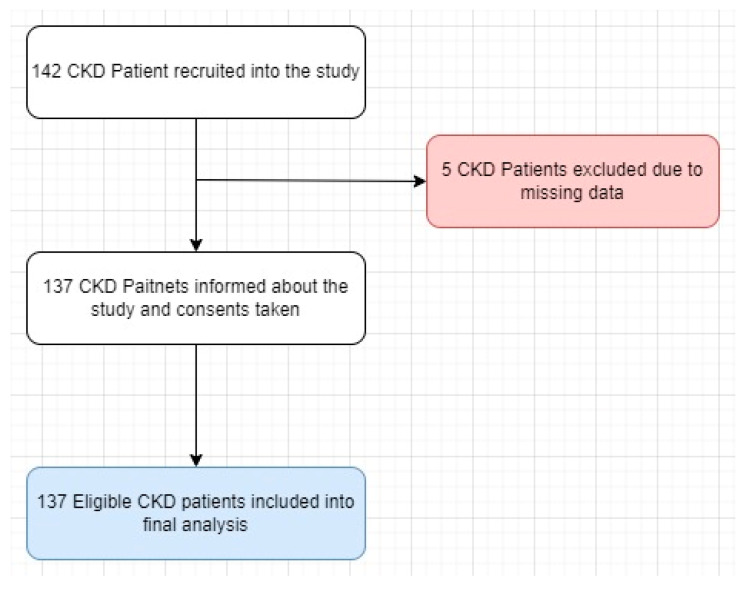
Study flow chart.

A total of 142 individuals were invited to participate, with 137 (96.48%) consenting to join the study (Figure 1). Patients with CKD were stratified based on the Kidney Disease: Improving Global Outcomes (KDIGO) classification. 

The characteristics of the Lexicomp Drug Interactions^®^ and Medscape^®^ used are given in Table 1. The distribution among groups was as follows: G3a (20 patients), G3b (40 patients), G4 (55 patients), and G5 (22 patients). Table 2 provides an overview of the baseline characteristics of the participants.

**Figure 2:**
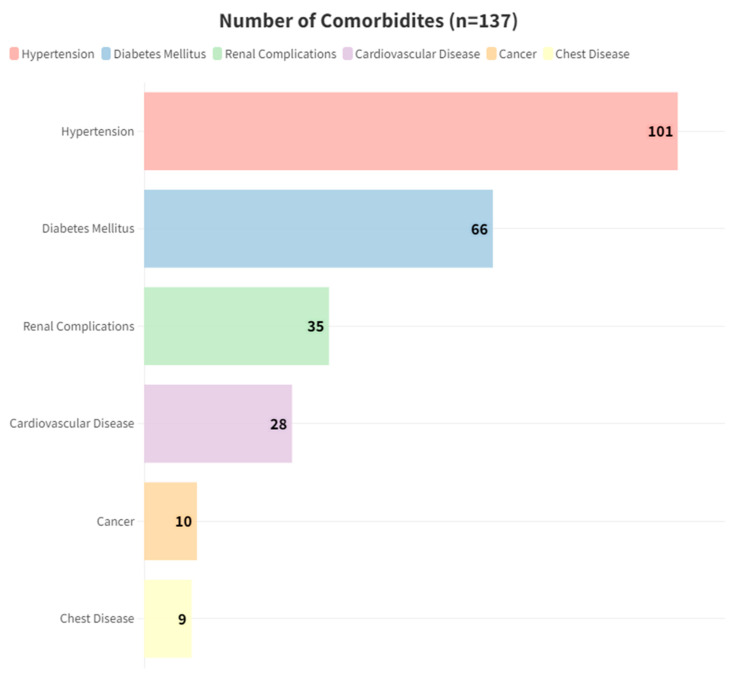
Number of most common comorbidities.

The mean age was 64.8 ± 14.59 years, with 80 (58.39%) individuals aged over 65 years, comprising 57 (41.6%) males and 80 (58.39%) females (Table 2). Twenty-four patients (17.5%) reported a history of smoking. The majority of patients had one or two comorbidities (155, 68%), with 80 (58.39%) having three or more comorbidities, and the median of comorbidities is 2 [1–3] (Table 2). Hypertension was the most prevalent comorbidity, affecting 101 (73.76%) individuals, followed by type II diabetes mellitus (66, 48.2%) and renal complications (35, 25.5%) (Figure 2).

A total of 88 participants (64.1%) were able to provide self-care for their health. Ambulatory blood pressure measurements and biochemical laboratory results related to renal disease are detailed in Table 2. The average number of medications per patient was 8.18 ± 3.84. Across the G3a, G3b, G4, and G5 groups categorized by renal impairment levels, the mean number of medications used were 8.6 ± 3.84, 7.25 ± 3.7, 8.4 ± 3.95, and 8.95 ± 3.79, respectively (*p* > 0.05) (Table 2).

**Figure 3:**
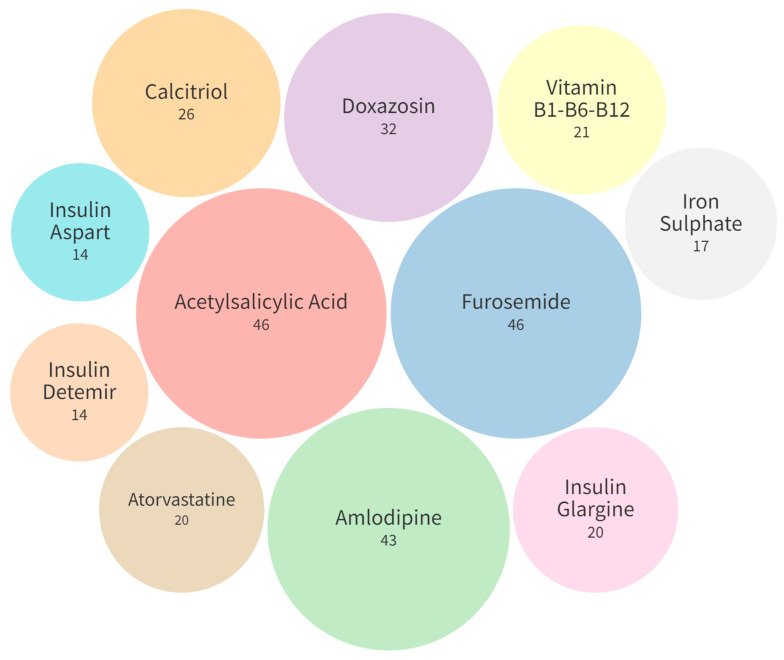
Most commonly prescribed medications (*n* = 137).

The most prescribed medicines were furosemide (46, 33.57%), acetylsalicylic acid (46, 33.57%), and amlodipine (43, 31.38%), as shown in Figure 3.

### Potential Drug–Drug Interactions

**Figure 4:**
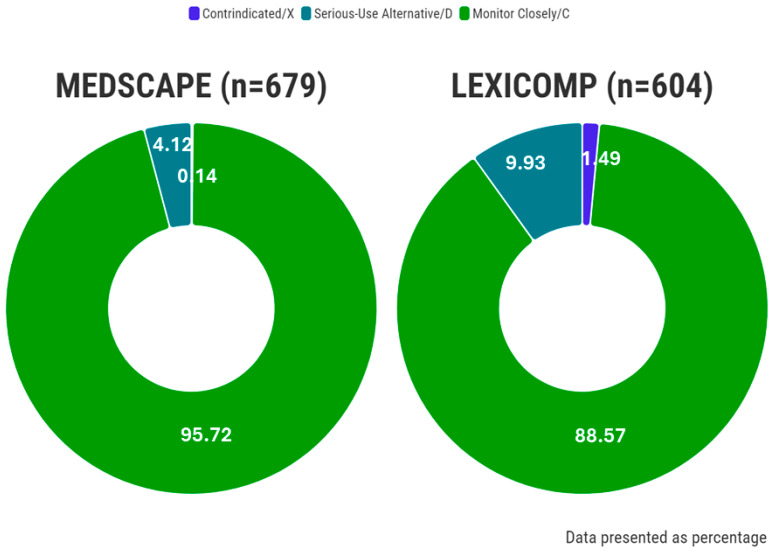
The ratio of interactions according to the different CDSS systems.

The total numbers of pDDIs requiring intervention identified by Medscape and Lexicomp were 679 and 604, respectively (Figure 4). Furthermore, the occurrences of contraindicated/X, serious-use alternative/D, monitor closely/C, levels of pDDIs per patient were observed as 0.01 ± 0.09/0.07 ± 0.25, 0.21 ± 0.47/0.44 ± 0.74, and, 4.77 ± 4.54/3.96 ± 4.10, respectively (Table 3). Notably, the proportion of significant pDDIs varied significantly between the Medscape and Lexicomp CDSS databases, accounting for 4.27% and 11.42%, respectively (*p* < 0.05).

According to the data from the Medscape and Lexicomp databases, the mean pDDIs per patient were 0.21 ± 0.49 and 0.5 ± 0.83, respectively. Similarly, across the G3a, G3b, G4, and G5 groups, the mean numbers of pDDIs per patient were 0.15 ± 0.37, 0.15 ± 0.36, 0.18 ± 0.47, and 0.45 ± 0.74, respectively (*p* > 0.05). The average duration of CKD was calculated as 6.48 ± 5.66 years. Again, across the G3a, G3b, G4, and G5 groups, the mean numbers of medications used were 8.6 ± 3.84, 7.25 ± 3.7, 8.4 ± 3.95, and 8.95 ± 3.79, respectively (*p* > 0.05).

Table 4 presents the evaluation of different CDSS systems in terms of inter-item and intra-class reliability using Cronbach’s α and Kendall’s W concordance analysis. The calculated Cronbach’s α value for CDSS was 0.315 (CI: 0.287–0.369, *p* = 0.028), indicating moderate internal consistency. Kendall’s W analysis revealed statistically significant agreement among two different CDSS systems regarding the number of pDDIs, rejecting the null hypothesis in favor of the alternative. However, two different CDSS systems exhibited statistically significant agreement in assessments with poor agreement (W = 0.073, *p* < 0.001). An ANOVA with Cochran’s Test further demonstrated a statistically significant difference among the CDSS systems, with a Cochran’s Q of 192.278 (*p* < 0.001). Fleiss’ kappa analysis also indicated a poor agreement between the CDSS performance (κ = 0.065, 95% CI: −0.065 to 0.196; *p* < 0.05).

Statistically significant inter-item correlations were observed among different CDSS systems. Specifically, the two-pair correlations between the two programs based on severity ranking revealed a Spearman’s rho correlation value of 0.187 for Lexicomp–Medscape (*p* < 0.001). These findings indicate a very low level of agreement between the two-pair correlations. The number of most common drug–drug interaction pairs is given in Table 5.

## 3. Discussion

### 3.1. Frequency and Severity of Potential Drug–Drug Interactions

In our study focusing on patients with CKD, we examined a total of 1121 medication orders prescribed to 137 individuals. Given the diverse range of medications typically administered to address various comorbidities, such as hypertension and diabetes mellitus, alongside other chronic conditions, the potential for pharmacokinetic (PK) and pharmacodynamic (PD) pDDIs is considerable. Consequently, a crucial aspect of therapy management involves a regular assessment of the presence of pDDIs, often facilitated by the use of screening tools designed for this purpose. To shed light on CDSS systems’ effectiveness, we conducted a comparative analysis of the Medscape and Lexicomp databases, specifically evaluating disparities in the identification and severity rating of pDDIs in patients with CKD.

Consistent with the findings in the literature, hypertension and diabetes emerges as the most prevalent comorbidities [13,14,15,16,17]. Our study revealed that 24 (17.51%) and 47 (34.31%) of the patients exhibited at least one or more serious-use alternative/D or contraindicated/X pDDIs as identified by Medscape and Lexicomp, respectively. Comparable rates in the literature spanned a range from 0.04% to 37.77% across various diseases [17,18,19,20,21,22,23]. Thus, our findings align closely with the existing literature, underscoring the prevalence and significance of pDDIs in patients with CKD.

Variations in PK and PD parameters frequently observed in patients with renal insufficiency pose a significant hurdle in pharmacological treatment [24]. Potential DDIs can be classified into PK interactions, altering drug disposition via coadministration, affecting absorption, distribution, plasma protein binding, metabolism, and excretion. Conversely, PD interactions modify drug effects at the site of action, impacting multiple physiological mechanisms. Moreover, pharmaceutical interactions, often underestimated, are commonplace, notably with simultaneous drug administration [25]. Potential drug–drug interactions can lead to medication-related issues due to PD or PK interactions. Among the most frequently encountered pDDIs in our study, acetylsalicylic acid–furosemide (*n* = 25), acetylsalicylic acid–metoprolol (*n* = 17), and acetylsalicylic acid–carvedilol (*n* = 13) represent common examples of PD interactions, whereas sodium bicarbonate–iron sulfate (*n* = 14) serves as an illustration of a pharmacokinetic interaction.

In the literature, commonly implicated drugs contributing to pDDIs in patients with CKD include loop diuretics, beta-blockers, oral iron supplements, proton pump inhibitors, and acetylsalicylic acid [18,26]. Previous studies have reported a moderate interaction between furosemide and aspirin, with a frequency ranging from 4.5% to 7.9% [19,27,28]. Clinical evidence suggests that their concurrent use can lead to a diminished diuretic and antihypertensive effect of furosemide, necessitating the monitoring of diuresis and creatinine clearance. The molecular mechanisms driving this interaction are likely attributed to the established effect of cyclooxygenase inhibitors, such as aspirin and other non-steroidal anti-inflammatory drugs (NSAIDs), on renal function. NSAIDs counteract the protective actions of prostaglandins in the kidneys, leading to compromised renal blood flow, glomerular filtration rate, and natriuresis. In CKD, where prostaglandin production is increased as a compensatory mechanism to improve organ perfusion, this interaction may be particularly relevant [29].

The concurrent administration of furosemide and ACE inhibitors, such as lisinopril, captopril, and enalapril, has been documented in numerous studies to induce severe postural hypotension, stemming from excessive vasodilation and relative intravascular volume depletion, as well as renal insufficiency due to reduced perfusion. This adverse effect is particularly notable following the initial dose, with frequency varying based on the degree of renal impairment and the specific ACE inhibitor utilized. The interaction between furosemide and lisinopril is reported with a frequency of approximately 7–9% [18,19,21,30], while the interaction between furosemide and enalapril occurs at a frequency of about 5–6%. Similarly, the interaction between furosemide and captopril is observed with a frequency of approximately 4–6% [18,21,27].

Omeprazole and pantoprazole, proton pump inhibitors (PPIs), have been noted to interact with oral iron supplements (OFSs) in various studies, albeit with a relatively low frequency ranging from 1% to 5% [17,18,21,22]. This moderate interaction, categorized as a type B/C pharmacokinetic, manifests rapidly as proton pump inhibitors elevate gastric pH, thereby impeding the absorption of OFS and consequently reducing non-heme iron bioavailability.

### 3.2. Comparison of Potential Drug–Drug Interaction Information from Different Sources

In our study, a poor level of concordance was observed between the Medscape and Lexicomp databases. However, in sub-analyses conducted at different levels of kidney failure, a slight level of positive concordance was obtained within the G3b kidney failure category. Comparable to the findings in the literature where various CDSS programs have been compared, the concordance levels among many programs align closely with those obtained in our study [13,14,16,23,31].

Predicting pDDIs is challenging, necessitating an expert-level understanding of pharmacology, pharmacogenetics, clinical practice across various specialties, and a thorough evaluation of evidence for potential side effects, including rare events [32,33]. Even when pDDIs are identified pharmacologically, determining their clinical impact can be difficult. To address these differences effectively, multidisciplinary teams comprising clinical pharmacists with extensive clinical experience need to collaborate in the detection and prevention of pDDIs.

The target users of drug interaction database programs vary widely in their understanding of pDDIs, including physicians, mid-level prescribers from diverse specialties, and pharmacists [34,35]. These programs utilize different sources of information, employ various rating criteria and procedures, and define different levels of acceptable risk. Over the past decade, researchers have consistently emphasized the lack of consistency among drug interaction database programs and compendia [36,37,38,39]. Despite efforts to enhance the selection of pDDI evidence, a broadly accepted standard for defining pDDI risk remains missing [37,38,40,41]. Given the complexity of the subject matter, disparities in results among drug interaction database programs are unsurprising and should be acknowledged. It is important to recognize the variability among these programs as a significant limitation. Relying solely on a single program for checking drug interactions could potentially endanger patients in certain cases [31].

There are several reasons contributing to the limited overlap and concordance observed among the two databases analyzed in our study. Firstly, the absence of a standardized definition for a pDDI results in varying interpretations of what constitutes a pDDI [41]. Different databases rely on diverse sources of information and establish distinct criteria for evidence levels necessary to define a pDDI for a specific drug combination. While case reports might suffice for one database, others may prioritize PK properties or studies on PD responses. Furthermore, the probability of a drug interacting with another often depends on various factors, including the interval between drug intake, dosage, and route of administration, which are not consistently accounted for across databases [31].

Concerning the severity rating of pDDIs, there exists no consistent definition of, for example, a mild pDDI. Additionally, differences in the completeness of drug and pDDI documentation across databases should be considered. Variances in update intervals mean that a particular pDDI might be documented differently across databases. Consequently, clinicians are currently advised to utilize multiple clinical decision support systems/drug–drug interaction databases and to consult clinical pharmacists to ensure that relevant pDDIs are not overlooked [7,42]. Severe pDDIs have the potential to induce life-threatening conditions, necessitating prompt medical intervention to avert serious consequences.

The prevalence of pDDIs in patients with CKD receiving hemodialysis and/or pharmacological treatment has been reported to range from 27.5% to 89.1% [28,43]. This broad range of probability of pDDIs is significant, with several factors potentially contributing to it, such as pre-existing comorbidities or complications, the quantity and types of prescribed medications per patient, and the stage of CKD.

Patients with kidney disease, as well as older individuals in general, may be particularly vulnerable to this burden, which is associated with an elevated risk of pDDIs and adverse drug-related events [20]. Despite the recognition of these high-risk conditions, the scientific literature on this topic remains limited. Surprisingly, systematic reviews and meta-analyses addressing the potential for DDIs in patients with polypharmacy are scarce. Considering the clinical characteristics of patients with CKD, relying solely on CDSS for the detection of pDDIs may increase the likelihood of errors. In patients with complex clinical profiles like CKD, establishing multidisciplinary healthcare teams involving clinical pharmacists can be beneficial for patient outcomes.

Consistent with previous studies, our findings indicate that online databases such as Micromedex^®^ and Lexicomp^®^ exhibit varying abilities to detect pDDIs in patients with CKD. The impact of pDDIs on mortality and morbidity is substantial, particularly among individuals with CKD, when compared with patients without CKD. Particularly in clinical settings lacking the presence of a clinical pharmacist, healthcare providers may rely on CDSS systems. However, it is more reliable to use different CDSS systems, yet they may yield conflicting results. Unfortunately, there is presently no integrated program that comprehensively encompasses the entirety of the medical literature and adequately addresses the aforementioned challenges. We observed a significant but poor agreement between the two online databases. The observed poor agreement on drug interactions identified by the online databases may be attributed to differences in the evaluation of pDDI evidence and varying severity categorizations [44].

In conclusion, our study revealed significant disparities in both the number and severity of pDDIs detected by two CDSS systems. These discrepancies pose a challenge for clinicians and may potentially result in suboptimal prescribing decisions. Efforts towards more efficient reporting and validation of these platforms could prove beneficial in addressing this issue and enhancing the quality of patient care.

### 3.3. Limitations

This study is subject to several limitations. Firstly, the participation of a limited number of patients within the planned timeframe may have constrained the depth of insights into the prevalence of drug interactions. Furthermore, the comparison focused solely on the severity categories of pDDIs, neglecting the exploration of other program attributes such as functionality and user-friendliness. Additionally, distinguishing adverse reactions resulting specifically from pDDIs versus those from individual drugs alone posed challenges in certain cases. To address this, consensus decisions were reached with the treating physician to ascertain the certainty of pDDI-related adverse reactions. A final limitation of our study pertains to the selection of a gold standard. While we utilized a conventional approach to compare CDSS systems, we did not evaluate their real-life impacts on decision-making processes.

## 4. Materials and Methods

### 4.1. Setting and Patient Characteristics

A prospective cross-sectional study was conducted at a university hospital in Istanbul, Turkey from September 2018 to April 2019. Patients with CKD visiting the nephrology outpatient clinic were recruited, and informed consent was obtained through signed consent forms. This study was approved by the local ethics committee (approval number 16/208) and adhered to the Strengthening the Reporting of Observational Studies in Epidemiology (STROBE) standards [45].

### 4.2. Sample Size

The sample size (SS) was determined using the following formula: SS = Z^2^ × *p* × (1 − *p*)/c^2^. Z represents the level of confidence (e.g., 1.96 for a 95% confidence level), *p* is the estimated percentage of selecting a choice (assumed as 50%), and c is the desired level of precision, set at 0.05. We calculated that a minimum of 95 patients would be required for inclusion in the analysis [46].

### 4.3. Data Acquisition and Evaluating the pDDIs

Patient follow-up occurred during nephrologist visits, where a comprehensive assessment was conducted by a multidisciplinary team comprising a nephrologist, a 5th-year pharmacy student, and a clinical pharmacist. Demographic data, including smoking and alcohol consumption, body mass index, co-medication use, herbal medicine and food supplement intake, and comorbidities, were collected. A reliable medication list for the past 6 months was compiled, and medical records were reviewed for information on medications, diagnoses, and treatment purposes (palliative/curative). The International Classification of Diseases 10th Revision (ICD-10) codes were used for diagnoses. Pharmacologically active components were counted, accounting for multiple ingredients in a single formulation.

This study assessed the capacity of two online databases, Lexicomp^®^ and Medscape^®^, to identify clinically relevant pDDIs in patients with CKD. Drug interactions were examined using the interaction checker tools provided by Lexicomp^®^ and Medscape^®^. The Lexicomp^®^ interaction checker classified interactions into five subgroups, offering recommendations for clinical approaches. A committee comprising a nephrologist, a 5th-year pharmacy student, and a clinical pharmacist analyzed Category D and X interactions, with corresponding recommendations such as ‘Consider Therapy Modification’ for Category D and ‘Avoid Combination’ for Category X. The Medscape^®^ interaction checker categorized interactions into five subgroups with recommendations, including none, minor, significant (monitor closely), serious (use alternative), and contraindicated. Clinically significant potential drug–drug interactions were considered for Lexicomp^®^, focusing on Categories X and D, and for Medscape^®^, focusing on the contraindicated and serious categories. The inter-rater reliability (Kappa Index) was determined to assess the agreement between each database and the gold standard.

### 4.4. Statistical Analysis

Continuous variables were reported as the mean ± standard deviation, while ordinal and nominal data were presented as number (*n*) and percentage (%). Baseline characteristics of the patients were described using proportions for dichotomous and categorical variables. Statistical differences between continuous variables were evaluated using Student’s *t*-test and non-parametric tests for repeated measures (Friedman Test). Categorical variables were compared using the chi-squared or Fisher exact tests.

Inter-item correlations among the software were analyzed using the Pearson correlation test. The association between pDDI software and the outcomes of three severity levels of interaction was assessed by evaluating each pDDI through Cronbach’s α, Kendall W, and ANOVA with Cochran’s test analysis. Statistical analysis was performed using IBM SPSS 26.0 and Jamovi, with a significance level set at *p* < 0.05.

## 5. Conclusions

pDDIs can be readily identified through the use of various interaction checker programs in routine clinical practice. However, the severity of these interactions may be categorized differently across different programs. Many elements influence the decision-making process in clinical practice, including clinician experience, contextual factors, and the availability of CDSS systems. While there are numerous resources available to assist clinicians, the lack of validation of various CDSS systems represents a significant gap in the field. This disparity may lead to issues where usability does not necessarily align with accuracy and availability.

This study identifies several areas for improvement in clinical decision-making processes. The findings highlight significant heterogeneity in the identification of drug–drug interactions among the various online databases compared. This discrepancy suggests that the information related to pDDIs in these databases may be sourced from different resources or based on different pieces of evidence.

Therefore, patient monitoring should be overseen by a multidisciplinary healthcare team, which includes a clinical pharmacist. The severity of potential drug interactions provided by the programs should be interpreted by professionals, taking into account unique patient characteristics such as age, comorbidities, and treatment dosage. Individualized decisions, such as considering alternative drug changes, dosage adjustments, or monitoring only, should be made in the treatment of patients with CKD. These efforts will be essential in optimizing patient care and minimizing the risks associated with pDDIs in this vulnerable population.

## Figures and Tables

**Table 1:** Comparing the primary attributes of various selected CDSS programs involves assessing their key features. * The availability of the software, whether solely accessible online requiring an internet connection (desktop or smartphone app), or offline, where internet connectivity is unnecessary, is a fundamental aspect to consider.

*p*DDI Software Program	Lexicomp Drug Interactions^®^	Medscape^®^
Language	English	English
Clinical effect	Yes	Yes
Online/offline *	Online	Online
Access/payment	Required	Not Required
Risk rating	Yes	Yes
Risk rating categories	X, D, C, B, A	Contraindicated, Serious-Use Alternative, Monitor Closely, Minor, None
Mechanism of interaction	Yes	Yes
Gives advice for clinical management	Yes	Yes
Reliability rating	Yes	No
Reliability rating categories	Good, Fair, Poor	No
Reference list	Yes	No
Date of last update	2 February 2024	Not Available
Source	Wolters Kluwer Clinical Drug Information	Medscape Publishers’ Circle

CDSS: clinical decision support system; pDDI: potential drug–drugs interaction.

**Table 2:** Characteristic parameters of participants (*n* = 137).

CKD Classification	G3a 45–59 mL/min/1.73 m^2^	G3b 30–44 mL/min/1.73 m^2^	G4 15–29 mL/min/1.73 m^2^	G5 <15 mL/min/1.73 m^2^	Total	*p*
Number of Patients (*n*, %)	20, 14.60	40, 29.20	55, 40.15	22, 16.06	137, 100	0.379
Gender (*n*, %)						0.043
Women	9, 6.6	20, 14.6	33, 24.1	18, 13.1	80, 58.4	
Men	11, 8	20, 14.6	22, 16.1	4, 2.9	57, 41.6	
Working Status (*n*, %)						0.236
Employed	8, 5.8	18, 13.1	17, 12.4	12, 8.8	55, 40.1	
Unemployed	12, 8.8	22, 16.1	38, 27.7	10, 7.3	82, 59.9	
Smoking (*n*, %)						0.375
Yes	4, 2.9	10, 7.3	8, 5.8	2, 1.5	24, 17.5	
No	16, 11.7	30, 21.9	47, 34.3	20, 14.6	113, 82.5	
Alcohol (*n*, %)						0.808
Yes	-	1, 0.7	1, 0.7	-	2, 1.5	
No	20, 14.6	39, 28.5	54, 39.4	22, 16.1	135, 98.5	
Age (years) (Mean ± SD)	62.35 ± 12.67	61.75 ± 15.85	68.36 ± 13.56	63.68 ± 15.38	64.8 ± 14.59	0.13
BMI (Mean ± SD)	30.52 ± 6.28	28.97 ± 5.46	28.05 ± 4.65	27.78 ± 5.85	28.63 ± 5.36	0.28
Weight (Kg)	88.8 ± 23.71	77.95 ± 14.28	74.85 ± 13.67	73.91 ± 16.04	77.64 ± 16.57	0.01
Height (Cm)	169.75 ± 10.18	164.25 ± 8.87	163.4 ± 10.57	163.23 ± 8.68	164.55 ± 9.9	0.008
No. of Comorbidities (Mean ± SD)	2.8 ± 0.89	2.23 ± 1.19	2.22 ± 1.13	2.05 ± 1.21	2.28 ± 1.14	0.15
No. of Drugs Used per Patient (Mean ± SD)	8.6 ± 3.84	7.25 ± 3.7	8.4 ± 3.95	8.95 ± 3.79	8.18 ± 3.84	0.30
No. of Comorbidities (Median, [IQR])	3, [2–3.75]	2, [1.25–3]	2, [1–3]	2, [1–3]	2, [1–3]	0.099
0 (*n*, %)	0, 0	3, 2.2	1, 0.7	1, 0.7	5, 3.65	
1 (*n*, %)	1, 0.7	7, 5.1	18, 13.1	7, 5.1	33, 24.08	
2 (*n*, %)	7, 5.1	15, 10.9	12, 8.8	8, 5.8	42, 30.65	
3 (*n*, %)	7, 5.1	9, 6.6	17, 12.4	3, 2.2	36, 26.27	
4 (*n*, %)	5, 3.6	5, 3.6	6, 4.4	2, 1.5	18, 13.13	
5 (*n*, %)	-	1, 0.7	1, 0.7	1, 0.7	3, 2.18	
No. of pDDIs (Mean ± SD) *	0.15 ± 0.37	0.15 ± 0.36	0.18 ± 0.47	0.45 ± 0.74	0.21 ± 0.49	0.088
No. of pDDIs (Mean ± SD) **	0.15 ± 0.49	0.75 ± 1.03	0.47 ± 0.77	0.45 ± 0.74	0.5 ± 0.83	0.059
Years with CKD (Mean ± SD)	5.95 ± 4.65	7.27 ± 7.15	5.98 ± 4.66	6.8 ± 5.91	6.48 ± 5.66	0.70
Capability of Self- Care (*n*, %)						0.333
Yes	16, 11.7	24, 17.5	36, 26.3	12, 8.8	88, 64.1	
No	4, 2.9	16, 11.7	19, 13.9	10, 7.3	49, 35.8	
Systolic Blood Pressure (mmHg)	135 ± 23.95	137 ± 21.51	93 ± 22.53	55 ± 24.59	136.69 ± 22.58	0.91
Diastolic Blood Pressure (mmHg)	70.5 ± 19.59	78.25 ± 12.79	75.55 ± 8.85	80 ± 15.12	76.31 ± 13.25	0.076
Albumin (g/dL) (Mean ± SD)	4.15 ± 0.26	4.1 ± 0.29	4.71 ± 4.72	3.99 ± 0.28	4.34 ± 3.02	0.71
Potassium (mmol/L) (Mean ± SD)	4.39 ± 1.12	4.68 ± 0.55	4.67 ± 0.58	4.78 ± 0.51	4.65 ± 0.67	0.26
Calcium (mg/dL) (Mean ± SD)	9.46 ± 0.48	9.08 ± 1.5	9.08 ± 0.51	8.77 ± 0.73	9.09 ± 0.95	0.13
Creatinine (mg/dL) (Mean ± SD)	1.24 ± 0.21	6.13 ± 27.71	2.5 ± 0.47	4.22 ± 1.1	3.65 ± 14.96	0.58
Ferritin (ng/mL) (Mean ± SD)	108.7 ± 101.11	122.89 ± 97.81	162.28 ± 203.14	202.22 ± 107.06	149.37 ± 152.63	0.13

* Number of contraindicated and serious pDDIs according to Medscape database; ** number of X- and D-level pDDIs according to Lexicomp database. CKD: chronic kidney disease; IQR: interquartile range; pDDI: potential drug–drug interactions; SD: standard deviation.

**Table 3:** Total and mean numbers of pDDIs detected by different clinical decision support software. Note: Calculations were made by taking into consideration only clinically significant interactions, including contraindicated, serious for Medscape, and X and D for Lexicomp.

	MEDSCAPE^®^			LEXICOMP^®^		
	Contraindicated	Serious-Use Alternative	Monitor Closely	X	D	C
G3a 45–59 mL/min/1.73 m^2^	0.05 ± 0.22	0.15 ± 0.37	3.55 ± 3.14	0.05 ± 0.22	0.30 ± 0.47	2.70 ± 3.25
G3b 30–44 mL/min/1.73 m^2^	-	0.15 ± 0.43	4.59 ± 4.27	0.03 ± 0.16	0.36 ± 0.74	3.49 ± 4.30
G4 15–29 mL/min/1.73 m^2^	-	0.24 ± 0.54	4.36 ± 3.94	0.07 ± 0.26	0.55 ± 0.83	3.87 ± 3.61
G5 <15 mL/min/1.73 m^2^	-	0.27 ± 0.46	7.23 ± 6.55	0.14 ± 0.35	0.45 ± 0.67	6.18 ± 4.93
Total (Mean ± SD) ^1^	0.01 ± 0.09	0.21 ± 0.47	4.77 ± 4.54	0.07 ± 0.25	0.44 ± 0.74	3.96 ± 4.10
Total No. of pDDIs (*n*, %)	1, 0.15	28, 4.12	650, 95.73	9, 1.49	60, 9.93	535, 88.58

^1^ Mean number of potential drug–drug interactions per patient; pDDI: potential drug–drug interaction; SD: standard deviation.

**Table 4:** Evaluation of different clinical decision support software (CDSS) programs via inter-item and intra-class reliability analysis.

**Inter-Item Correlation Matrix**			Medscape		***p*** **-value**
Lexicomp			0.187		<0.001
**Intraclass Correlation Coefficient**					
	Intraclass Correlation Coefficient		95% Confidence Interval		
			Lower Bound	Upper Bound	***p*** **-value**
Cronbach’s α	0.315		0.287	0.369	0.028
**Kendall Coefficient of Concordance of Lexicomp and Medscape Software**					
	**Kendall W**	**Chi-Square**	**Strength of agreement**	***p*** **-value**	***n***
Overall	0.073	9.981	Poor	<0.001	137
G3a 45–59 mL/min/1.73 m^2^	0.173	0.200	Poor	0.655	20
G3b 30–44 mL/min/1.73 m^2^	0.268	10.714	Slight	0.001	40
G4 15–29 mL/min/1.73 m^2^	0.091	5.000	Poor	0.025	55
G5 <15 mL/min/1.73 m^2^	0.006	0.143	Poor	0.705	22

**Table 5:** Most common drug–drug interactions according to Lexicomp and Medscape CDSS.

Drug-Drug Interactions	LEXICOMP		MEDSCAPE		Explanation	Severity/Reliability Rating
	Severity	*n*	Severity	*n*		
Acetylsalicylic Acid–Furosemide	C	25	Monitor closely	25	Acetylsalicylic acid may reduce diuretic effect of furosemide. May increase serum concentration.	Moderate/Good
Acetylsalicylic Acid–Metoprolol	No interactions		Monitor closely	17	Acetylsalicylic acid reduces PD antagonism effect of metoprolol. Both increase serum potassium levels.	NA
Allopurinol–Furosemide	C	19	No interactions		Furosemide may increase toxic effect of allopurinol. Increases serum concentration.	Moderate/Fair
Sodium bicarbonate–Iron Sulfate	D	14	Monitor closely	14	Sodium bicarbonate reduces absorption of iron sulfate.	Minor/Fair
Acetylsalicylic Acid–Carvedilol	No interactions		Monitor closely	13	Acetylsalicylic acid decreases effects of carvedilol by PD antagonism.	NA
Furosemide–Doxazosin	C	11	No interactions		Acetylsalicylic acid may increase hypotensive effect of doxazosin.	Moderate/Fair
Furosemide–Hydrochlorothiazide	C	11	Monitor closely	11	Furosemide may enhance hypotensive effect of antihypertensive agents.	Moderate/Fair
Metoprolol–Furosemide	C	11	Monitor closely	11	Metoprolol increases serum potassium levels, decreases furosemide.	Moderate/Fair
Metoprolol–Doxazosin	C	10	Monitor closely	10	Metoprolol may enhance orthostatic hypotensive effect of doxazosin.	Moderate/Fair
Acetylsalicylic Acid–Doxazosin	No interactions		Monitor closely	11	Acetylsalicylic acid reduces effect of doxazosin by PD antagonism.	NA
Metformin–Hydrochlorothiazide	C	9	Minor	9	Hydrochlorothiazide may reduce therapeutic effect of metformin.	Moderate/Fair
Acetylsalicylic Acid–Hydrochlorothiazide	No interactions		Monitor closely	11	Acetylsalicylic acid increases serum potassium levels, decreases hydrochlorothiazide.	NA
Acetylsalicylic Acid–Valsartan	No interactions		Monitor closely	10	PD synergism/both increase serum potassium levels.	NA
Carvedilol–Valsartan	No interactions		Monitor closely	10	Pharmacodynamic synergism.	NA
Acetylsalicylic Acid–Clopidogrel	C	9	Monitor closely	8	Both enhance antiplatelet effects of each other.	Moderate/Fair
Metoprolol–Doxazosin	B	9	Monitor closely	9	Metoprolol may enhance hypotensive effect of doxazosin.	Minor/Fair
Insulin Aspart–Furosemide	C	8	No interactions		Furosemide reduces therapeutic effect of insulin.	Moderate/Fair
Acetylsalicylic Acid–Insulin Glargine	C	8	Monitor closely	8	Acetylsalicylic acid may increase effect of insulin glargine.	Moderate/Fair
Sodium bicarbonate–Allopurinol	No interactions		Monitor closely	9	Sodium bicarbonate reduces allopurinol levels by inhibition of gastrointestinal absorption.	NA
Metoprolol–Amlodipine	No interactions		Monitor closely	8	Doxazosin and amlodipine both increase anti-hypertensive channel blocking.	NA
Acetylsalicylic Acid–Clopidogrel	C	8	Monitor closely	8	Agents with antiplatelet properties may enhance antiplatelet effect of other agents with antiplatelet properties.	Moderate/Fair
Doxazosin–Amlodipine	B	8	Monitor closely	8	Antihypertensive agents may enhance hypotensive effect of doxazosin.	Minor/Fair
Doxazosin–Carvedilol	B	8	Monitor closely	8	Antihypertensive agents may enhance hypotensive effect of doxazosin.	Minor/Fair
Nebivolol–Acetylsalicylic Acid	No interactions		Monitor closely	7	Acetylsalicylic acid decreases effects of nebivolol by PD antagonism.	NA
Nebivolol–Hydrochlorothiazide	No interactions		Monitor closely	7	Nebivolol increases and hydrochlorothiazide decreases serum potassium.	NA
Furosemide–Carvedilol	C	7	Monitor closely		Furosemide may enhance hypotensive effect of antihypertensive agents.	Moderate/Fair
Carvedilol–Hydrochlorothiazide	No interactions		Monitor closely	7	Carvedilol increases serum potassium levels, decreases hydrochlorothiazide.	NA
Sodium bicarbonate–Nebivolol	No interactions		Monitor closely	7	Sodium bicarbonate reduces nebivolol levels by inhibition of gastrointestinal absorption.	NA
Pantoprazole–Iron Sulfate	B	7	Monitor closely	7	Inhibitors of proton pump may decrease absorption of iron preparations.	Minor/Fair
Insulin Glargine–Furosemide	C	7	No interactions		Furosemide reduces therapeutic effect of allopurinol.	Moderate/Fair
Iron sulfate–Levothyroxine	D	6	Monitor closely	6	Iron II glycine sulfate may decrease serum concentration of levothyroxine.	Moderate/Good
Metformin–Furosemide	C	6	Minor	6	Furosemide may reduce therapeutic effect of metformin.	Moderate/Fair
Insulin Glargine–Insulin Aspart	C	6	No interactions		Insulin glargine increases hypoglycemic effect of insulin aspartate.	Moderate/Fair
Pantoprazole–Clopidogrel	C	6	Monitor closely	6	Pantoprazole reduces serum concentration of clopidogrel.	Major/Fair
Insulin Glargine–Metformin	C	5	Monitor closely	5	Metformin increases hypoglycemic effect of insulin glargine.	Moderate/Fair
Insulin Aspart–Linagliptin	D	4	No interactions		Linagliptin may increase hypoglycemic effect of insulin aspart.	Moderate/Fair
Acetylsalicylic Acid–Escitalopram	C	4	Monitor closely	4	Escitalopram increases antiplatelet effect of acetylsalicylic acid.	Moderate/Fair
Amlodipine–Clopidogrel	C	4	Monitor closely	4	Amlodipine reduces therapeutic effect of clopidogrel.	Moderate/Fair
Gliclazide–Furosemide	C	4	No interactions		Gliclazide may reduce therapeutic effect of furosemide.	Moderate/Fair

PD: Pharmacodynamic.

## Data Availability

The data will be shared upon request from the corresponding author.
